# Vaccinia Virus among Domestic Dogs and Wild Coatis, Brazil, 2013–2015

**DOI:** 10.3201/eid2412.171584

**Published:** 2018-12

**Authors:** Galileu Barbosa Costa, Lara Ribeiro de Almeida, Aline Gabriele Ribeiro Cerqueira, Wander Ulisses Mesquita, Jaqueline Silva de Oliveira, Júlia Bahia Miranda, Ana Teresa Saraiva-Silva, Jônatas Santos Abrahão, Betânia Paiva Drumond, Erna Geessien Kroon, Pedro Lúcio Lithg Pereira, Danielle Ferreira de Magalhães Soares, Giliane de Souza Trindade

**Affiliations:** Universidade Federal de Minas Gerais, Belo Horizonte, Brazil (G.B. Costa, L.R. de Almeida, A.G.R. Cerqueira, J.S. de Oliveira, J.B. Miranda, A.T. Saraiva-Silva, J.S. Abrahão, B.P. Drumond, E.G. Kroon, P.L.L. Pereira, D.F. de Magalhães Soares, G. de Souza Trindade);; Universidade Federal de Ouro Preto, Ouro Preto, Brazil (W.U. Mesquita)

**Keywords:** Orthopoxvirus, vaccinia virus, urban domestic dogs, wild coatis, public health, emerging virus, viruses, Brazil, zoonoses

## Abstract

To determine their potential role as a source of human infection, we tested domestic dogs (urban) and wild coatis (wild) in Brazil for vaccinia virus. Our findings of positive neutralizing antibodies and quantitative PCR results for 35/184 dogs and 13/90 coatis highlight a potential public health risk.

Since smallpox was declared eradicated in 1980, after a massive effort led by the World Health Organization, other orthopoxviruses have gained notoriety as zoonotic agents worldwide (*1*). Over the past 17 years in Brazil, many zoonotic outbreaks of vaccinia virus (VACV) infection have been recorded throughout the country, becoming a burden for the dairy industry and public health (*2*). The most affected hosts during outbreaks are dairy cattle and humans (*2*). Recent studies assessing the role of wildlife in the maintenance cycle of VACV in nature have corroborated previous findings that rodents and marsupials serve as links between natural and anthropic environments (*2**–**4*).

Indeed, the increased frequency of reported VACV detection in several species of mammals points toward new insights into the circulation and maintenance of VACV in wild (forest) and rural (farm) environments (*2**,**5**–**8*). Studies conducted in Latin America suggest that wildlife, especially small and medium-sized mammals, plays a role in virus transmission and maintenance of orthopoxviruses in nature (*9*). Furthermore, some studies have shown the presence of VACV in urban environments, emphasizing the risks for humans (especially those not vaccinated against smallpox) (*10**,**11*).

To determine the potential role of domestic and wild animals as a source of VACV infection for humans, we investigated VACV circulation among domestic dogs and wild coatis, animals that live at the intersection of urban and wild environments in Brazil. The capture of wild animals was authorized by the Brazilian Institute of Environment and Renewable Natural Resources, and the study was approved by the Ethics Committee in Animal Experimentation of Universidade Federal de Minas Gerais.

## The Study

**Figure 1:**
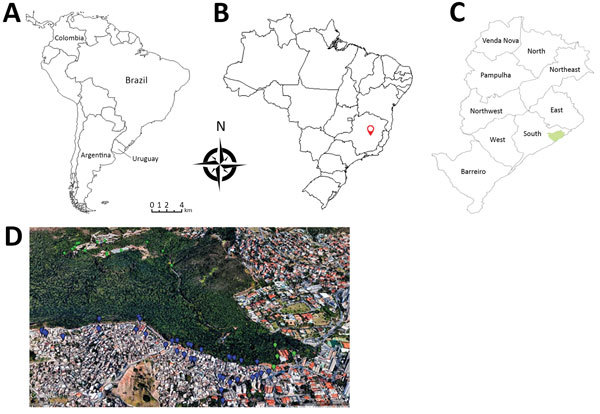
Area of study of vaccinia virus among domestic dogs and wild coatis, Brazil, 2013–2015. A) Countries in South America where vaccinia virus has been detected in recent years. B) Belo Horizonte (red locator), located in Minas Gerais state, Brazil. C) Regions of Belo Horizonte; green indicates area in wild environment where coatis were captured. D) Google Earth map from 2017 of studied area, showing details of the wild and urban environments. Green dots indicate where coatis were captured; blue dots indicate where dogs were sampled. Source: https://www.google.com/earth.

We analyzed serum and anal swab samples collected during 2013–2015 from 184 domestic dogs and 90 wild coatis in the city of Belo Horizonte (19°55′15′′S, 43°56′16′′W) in the state of Minas Gerais, Brazil (Figure 1). Swab samples of lesions, if present, were also collected. To determine the presence of neutralizing antibodies in serum, we used an orthopoxvirus plaque reduction neutralization test as previously described (*12*). Serum titers were defined as the highest dilutions that inhibited >70% of virus plaques compared with negative controls.

To detect VACV DNA from serum and anal swab samples, we performed real-time PCR targeting the C11R or A56R gene (*12*). We directly sequenced A56R fragments in both orientations and in triplicate by using the ABI3130 platform (Applied Biosystems, Waltham, MA, USA). Sequences were aligned with other reference sequences from GenBank by using MEGA 7.0 (http://www.megasoftware.net). Statistical analyses were conducted by using Epi Info software version 7.2.1.0 (https://www.cdc.gov/epiinfo); χ^2^ and Fisher exact tests were applied with significance set at 5%. We also calculated relative odds ratios (ORs) and 95% CIs.

We detected orthopoxvirus neutralizing antibodies in 35 dogs (prevalence rate 19.0%, 95% CI 14.0%–25.5%; titers 100–400 neutralizing units/mL) and in 13 coatis (prevalence rate 14.4%, 95% CI 8.5%–23.3%; titers 100–800 neutralizing units/mL) (Table 1). Univariate analyses indicated significant associations between presence of neutralizing antibodies and the following: male dogs (OR 2.6; p = 0.02), dogs 6–10 years of age (OR 5.2; p = 0.04), coatis captured in 2013 (OR 11.2; p = 0.002), juvenile coatis (<1 y of age) (OR 35; p = 0.001), and adult coatis (>2 y of age) (OR 5.1; p = 0.04).

**Table 1:** Associations between neutralizing antibodies against *Orthopoxvirus* and demographic characteristics of domestic dogs and wild coatis, Belo Horizonte, Brazil, 2013–2015*

Variable	No. (%)†	No. (%) positive‡	No. (%) negative‡	p value	Odds ratio (95% CI)
Domestic dogs					
Year of sampling					
2014	123 (66.8)	24 (19.5)	99 (80.5)		
2015	61 (33.2)	11 (18.0)	50 (82.0)	1.00	
Sex					
F	85 (46.4)	23 (27.1)	62 (72.9)	Reference	
M	96 (52.5)	12 (12.5)	84 (87.5)	**0.02**	**2.6 (1.2–5.6)**
Age, y					
<1	24 (13.1)	7 (29.2)	17 (70.8)	Reference	
2–5	82 (44.8)	16 (19.5)	66 (80.5)	0.4	
6–10	41 (22.4)	3 (7.3)	38 (92.7)	**0.04**	**5.2 (1.2–22.6)**
>10	18 (9.8)	4 (22.2)	14 (87.8)	0.9	
Size					
Small	75 (41.0)	13 (18.8)	56 (81.2)	Reference	
Medium	69 (37.7)	11 (20.0)	44 (80.0)	1.00	
Large	30 (16.4)	4 (14.3)	24 (85.7)	0.8	
Confinement status					
Always inside home	41 (22.4)	8 (19.5)	33 (80.5)	Reference	
Always in backyard	115 (62.8)	18 (15.6)	97 (84.3)	0.7	
Home and backyard	25 (13.7)	9 (36.0)	16 (64.0)	0.2	
Outdoors access†					
Yes	83 (45.3)	19 (22.9)	64 (77.1)	0.3	
No	98 (53.6)	16 (16.3)	82 (83.7)	Reference	
Access to MMP					
Yes	18 (9.8)	6 (33.3)	12 (66.7)	0.2	
No	101 (55.2)	18 (17.8)	83 (82.2)	Reference	
Wild coatis					
Year of capture					
2013	57 (52.8)	12 (21.0)	34 (59.6)	**0.002**	**14.8 (1.8–119.8)**
2014	51 (47.2)	1 (1.9)	42 (82.3)	Reference	
Sex					
F	64 (59.3)	10 (15.6)	44 (68.7)	Reference	
M	44 (40.7)	3 (6.8)	32 (72.7)	0.3	
Age group					
Juvenile, <1 y	44 (40.7)	1 (2.3)	35 (79.5)	Reference	
Subadult, 1–2 y	18 (16.7)	5 (27.8)	10 (55.6)	**0.01**	**0.05 (0.006–0.5)**
Adult, >2 y	46 (42.6)	7 (15.2)	31 (67.4)	**0.04**	**5.1 (1.2–22.6)**

*Totals may not add up to 100% because of missing information. Boldface indicates significance; odds ratios are shown only for significant results. MMP, Mangabeiras Municipal Park. †Includes access beyond backyard. ‡By plaque reduction neutralization test.

Samples from all seropositive animals were submitted for quantitative PCR (qPCR) to detect VACV DNA (Table 2). Overall, serum samples from 7 dogs and 6 coatis were positive for the C11R gene; of these, anal swab samples were positive for this gene for 3 dogs and 4 coatis. Samples from the C11R-positive animals were submitted for another qPCR targeting the A56R gene. Serum samples were positive for the A56R gene for 5 dogs and 4 coatis; of these, anal swab samples were positive for A56R for 1 dog and 3 coatis. No lesion swab samples were positive by qPCR for both C11R and A56R genes.

**Table 2:** Diagnostic results for 7 domestic dogs and 6 wild coatis with neutralizing antibodies for vaccinia virus*,* Belo Horizonte, Brazil, 2031–2015*

Animal	PRNT_70_ titer (NU/mL)	qPCR C11R			qPCR A56R		Strain
Serum sample	Anal swab sample	Serum sample	Anal swab sample
Dog 2	1:40 (100)	+	–		+	–	Group 1
Dog 58	1:80 (200)	+	+		+	–	Group 1
Dog 41	1:40 (100)	+	–		–	–	
Dog 77	1:80 (200)	+	+		+	–	Group 1
Dog 86	1:40 (100)	+	–		–	–	
Dog 121	1:160 (400)	+	–		+	–	Group 1
Dog 128	1:160 (400)	+	+		+	+	Group 2
Coati 5	1:40 (100)	+	+		+	+	Group 2
Coatis 17	1:40 (100)	+	–		–	–	
Coatis 27	1:80 (200)	+	–		–	–	
Coatis 39	1:160 (400)	+	+		+	+	Group 2
Coatis 48	1:40 (100)	+	+		+	–	Group 1
Coatis 50	1:80 (200)	+	+		+	+	Group 2

*NU, neutralizing units; PRNT_70_, 70% plaque reduction neutralization test; qPCR, quantitative PCR.

Alignment of the amplified A56R fragments showed high similarity to the homologous gene of VACV isolates from Brazil (Technical Appendix Figure). Furthermore, 5 sequenced samples (from 4 dogs and 1 coatis) showed an 18-nt signature deletion, which is present in sequences of mouse nonvirulent VACV strains from Brazil (group 1 VACV). This deletion was not present in samples from 4 animals (1 dog and 3 coatis), grouping with mouse virulent VACV strains from Brazil (group 2 VACV).

## Conclusions

We assessed VACV exposure of 2 interacting species of animal: domestic dogs from an urban area and coatis from a bordering wild area. In contrast to VACV infections, human cowpox virus infections have mostly occurred in urban areas of Europe. Cowpox virus is transmitted to humans mainly by domestic cats that are in contact with rodents, the natural cowpox virus reservoirs (*13*). However, some authors have hypothesized that domestic dogs could be implicated in the transmission cycle of VACV, acting as a link between the natural reservoirs and humans in urban environments (*14**,**15*). Indeed, our molecular findings support exposure and possible VACV infection of these animals, thereby indicating that they are a potential source of VACV exposure for humans in urban areas. 

The seroprevalence of orthopoxvirus neutralizing antibodies in dogs in Brazil has been described. Peres et al. found that, along with other farm animals, 22.8% of 114 dogs tested were seropositive for orthopoxviruses (*7*); this seroprevalence differs from that observed in our study, which was 3.8% lower. In addition, most animals tested by Peres et al. were from rural areas where no bovine vaccinia outbreaks had been officially reported, and 96% of farmers declared that their domestic animals have contact with wild animals (*9*). These findings indicate that dogs could be exposed to VACV through contact with wild animals, corroborating our hypothesis.

We also detected orthopoxvirus neutralizing antibodies in wild coatis, which is consistent with results of a previous study that described the seroprevalence of orthopoxviruses in procyonids from Mexico (*9*). Our detection of VACV DNA in anal swab samples from coatis indicate that these animals could act as a source of virus for domestic dogs and humans and serve as a link between wild and urban environments. However, future studies to determine if viable virus is shed are needed to confirm this possibility.

**Figure 2:**
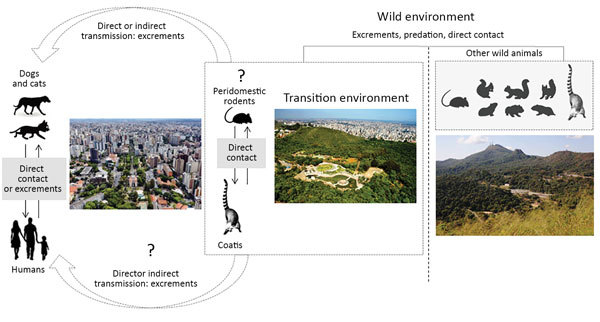
A hypothetical model developed to visualize the role of domestic animals and wildlife in the natural cycle of vaccinia virus (VACV). The model illustrates the dynamics of VACV circulation in urban and wild areas of Brazil. In urban areas, wild coatis could promote the transmission of VACV between domestic animals or humans because they are in direct contact with domestic dogs and circulate among urban residences. Domestic dogs could also promote the transmission of VACV to humans because of direct contact or possibly indirect contact thought contaminated feces. In the wild environment, coatis can interact with other mammals such as wild rodents, which are believed to be VACV reservoirs, and acquire the infection (this potential interaction is still under investigation).

To impart information about the role of domestic animals and wildlife in the natural cycle of VACV, we developed a hypothetical model based on previous studies (*2**,**3**,**5*), which could illustrate the dynamics of VACV circulation in urban areas (Figure 2). Because coatis can circulate in wild environments and surrounding urban areas, they could act as a bridge promoting the transmission of VACV between wild animals (mainly rodents) and dogs or humans. Domestic dogs could transmit VACV directly to humans through close contact or indirectly thought contaminated feces (Figure 2). These data raise questions about VACV circulation in Brazil and open discussions about the role of dogs and coatis in the VACV epidemiologic cycle.

Technical Appendix Nucleotide sequences of vaccinia virus A56R gene from domestic dogs and wild coatis, Brazil, 2013–2015, compared with sequences from other orthopoxviruses.

## References

[1] Shchelkunov SN. An increasing danger of zoonotic orthopoxvirus infections. PLoS Pathog. 2013;9:e1003756. 10.1371/journal.ppat.1003756 24339772 PMC3855571

[2] Oliveira JS, Figueiredo PO, Costa GB, Assis FL, Drumond BP, da Fonseca FG, et al. Vaccinia virus natural infections in Brazil: the good, the bad, and the ugly. Viruses. 2017;9:E340. 10.3390/v9110340 29140260 PMC5707547

[3] Miranda JB, Borges IA, Campos SPS, Vieira FN, de Ázara TMF, Marques FA, et al. Serologic and molecular evidence of vaccinia virus circulation among small mammals from different biomes, Brazil. Emerg Infect Dis. 2017;23:931–8. 10.3201/eid2306.161643 28518030 PMC5443434

[4] Peres MG, Bacchiega TS, Appolinário CM, Vicente AF, Mioni MSR, Ribeiro BLD, et al. Vaccinia virus in feces and urine of wild rodents from São Paulo state, Brazil. Viruses. 2018;10:E51. 10.3390/v10020051 29360742 PMC5850358

[5] Abrahão JS, Guedes MIM, Trindade GS, Fonseca FG, Campos RK, Mota BF, et al. One more piece in the VACV ecological puzzle: could peridomestic rodents be the link between wildlife and bovine vaccinia outbreaks in Brazil? PLoS One. 2009;4:e7428. 10.1371/journal.pone.0007428 19838293 PMC2758550

[6] Abrahão JS, Silva-Fernandes AT, Lima LS, Campos RK, Guedes MI, Cota MM, et al. Vaccinia virus infection in monkeys, Brazilian Amazon. Emerg Infect Dis. 2010;16:976–9. 10.3201/eid1606.091187 20507750 PMC3086250

[7] Peres MG, Bacchiega TS, Appolinário CM, Vicente AF, Allendorf SD, Antunes JM, et al. Serological study of vaccinia virus reservoirs in areas with and without official reports of outbreaks in cattle and humans in São Paulo, Brazil. Arch Virol. 2013;158:2433–41. 10.1007/s00705-013-1740-5 23760628 PMC3830743

[8] Peres MG, Barros CB, Appolinário CM, Antunes JM, Mioni MS, Bacchiega TS, et al. Dogs and opossums positive for vaccinia virus during outbreak affecting cattle and humans, São Paulo state, Brazil. Emerg Infect Dis. 2016;22:271–3. 10.3201/eid2202.140747 26812352 PMC4734541

[9] Gallardo-Romero NF, Aréchiga-Ceballos N, Emerson GL, Martínez-Martínez FO, Doty JB, Nakazawa YJ, et al. Endemic orthopoxvirus circulating in procyonids in Mexico. J Wildl Dis. 2016;52:609–15. 10.7589/2015-10-291 27224209 PMC6379897

[10] Dutra LA, de Freitas Almeida GM, Oliveira GP, Abrahão JS, Kroon EG, Trindade GS. Molecular evidence of Orthopoxvirus DNA in capybara (*Hydrochoerus hydrochaeris*) stool samples. Arch Virol. 2017;162:439–48. 10.1007/s00705-016-3121-3 27771792

[11] Costa GB, Miranda JB, Almeida GG, Silva de Oliveira J, Pinheiro MS, Gonçalves SA, et al. Detection of vaccinia virus in urban domestic cats, Brazil. Emerg Infect Dis. 2017;23:360–2. 10.3201/eid2302.161341 28098542 PMC5324812

[12] Geessien Kroon E, Santos Abrahão J, de Souza Trindade GS, Pereira Oliveira G, Moreira Franco-Luiz AP, Barbosa Costa G, et al. Natural vaccinia virus infection: diagnosis, isolation, and characterization. Curr Protoc Microbiol. 2016;42:14A.5.1–14A.5.43. 10.1002/cpmc.13 27517335

[13] Essbauer S, Pfeffer M, Meyer H. Zoonotic poxviruses. Vet Microbiol. 2010;140:229–36. 10.1016/j.vetmic.2009.08.026 19828265 PMC9628791

[14] Smith KC, Bennett M, Garrett DC. Skin lesions caused by orthopoxvirus infection in a dog. J Small Anim Pract. 1999;40:495–7. 10.1111/j.1748-5827.1999.tb03003.x 10587928

[15] von Bomhard W, Mauldin EA, Breuer W, Pfleghaar S, Nitsche A. Localized cowpox infection in a 5-month-old Rottweiler. Vet Dermatol. 2011;22:111–4. 10.1111/j.1365-3164.2010.00923.x 20735769

