# Supplementary material for: Vaccinia Virus among Domestic Dogs and Wild Coatis, Brazil, 2013–2015
Source: Emerg Infect Dis. 2018 Dec;24(12):2338–42. doi: 10.3201/eid2412.171584 (PMC6256396; doi:10.3201/eid2412.171584)
Supplement: Technical Appendix — Nucleotide sequences of vaccinia virus A56R gene from domestic dogs and wild coatis, Brazil, 2013–2015, compared with sequences from other orthopoxviruses. [file 17-1584-Techapp-s1.pdf]

Article DOI: https://doi.org/10.3201/eid2412.171584

Vaccinia Virus among Domestic Dogs and Wild Coatis, Brazil, 2013–2015

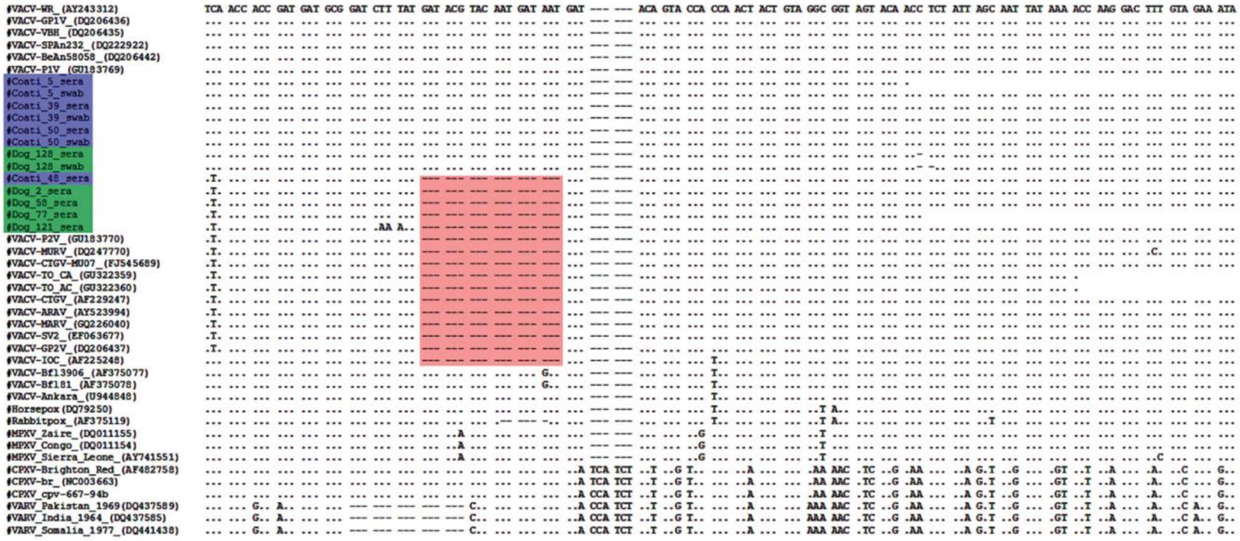

Technical Appendix Figure. Nucleotide sequence of the vaccinia virus A56R (hemagglutinin) gene found in domestic dogs (green) and wild coatis (purple) compared with homologous sequences of several other orthopoxviruses. Strains containing the conserved deletion region (red) were grouped with other vaccinia viruses (groups 1 and 2) isolated in Brazil.
